# Regional Variations in Physical Fitness and Activity in Healthy and Overweight Ecuadorian Adolescents

**DOI:** 10.3390/children5080104

**Published:** 2018-08-02

**Authors:** Cheryl A. Howe, Sharon Casapulla, Jay H. Shubrook, Pablo Lopez, Mario Grijalva, Darlene E. Berryman

**Affiliations:** 1 School of Applied Health Sciences and Wellness, Ohio University, Athens, OH 45701, USA; berrymad@ohio.edu; 2 The Diabetes Institute, Heritage College of Osteopathic Medicine, Ohio University, Athens, OH 45701, USA; casapull@ohio.edu (S.C.); Jay.Shubrook@tu.edu (J.H.S.); 3 Department of Family Medicine, Heritage College of Osteopathic Medicine, Ohio University, Athens, OH 45701, USA; 4 Office of Rural and Underserved Programs, Heritage College of Osteopathic Medicine, Ohio University, Athens, OH 45701, USA; 5 Infectious and Tropical Disease Institute, Department of Biomedical Sciences, Heritage College of Osteopathic Medicine, Ohio University, Athens, OH 45701, USA; grijalva@ohio.edu; 6 Department of Primary Care, Touro University College of Osteopathic Medicine, Vallejo, CA 90720, USA; 7 Center for Research on Health in Latin America, School of Human Nutrition, Pontifical Catholic University of Ecuador, Quito 170109, Ecuador; pelopez@puce.edu.ec

**Keywords:** cardiorespiratory fitness, youth, pediatric obesity, Ecuador, rural, urban

## Abstract

*Background:* Insufficient physical activity (PA) and excessive sedentary behavior (SB) are the main contributors to adolescent obesity. However, it is uncertain whether recent economic growth and urbanization in Ecuador are contributing to an obesogenic environment. This study assessed the relationships among fitness, PA, SB, and perceived social support for PA in adolescents from urban (Quito) and rural (Loja) Ecuador. *Methods:* Fitness was estimated using 3-min step test and PA and SB participation and social support for PA were self-reported in 407 adolescents. *T*-tests and analysis of variance assessed differences by sex, obesity status, and region of Ecuador. Pearson correlations assessed relationships among PA, SB, fitness, and social support. *Results:* Males and rural adolescents (48.3 ± 9.4 and 47.1 ± 9.6 mL/kg/min) were more fit than females and urban adolescents (41.1 ± 7.5 and 39.7 ± 6.1 mL/kg/min). Fitness was negatively correlated with obesity only in rural Ecuador. Few adolescents reported ≥60 min/day of PA (8.4%) or ≤2 h/day of SB (30.2%), with greater SB participation in rural Ecuador. Weak correlations were observed among fitness, PA, SB, and parental/peer support for PA (*r* = −0.18 to 0.19; *p* < 0.05). *Conclusion:* While fitness varied by sex, weight status, and region, SB participation and parent/peer support for PA, not PA participation itself, predicted fitness in rural Ecuadorean adolescents.

## 1. Introduction

Childhood obesity continues to be a growing global problem, rising tenfold in the past four decades with more than 124 million youth (5–19 years) classified as obese in 2016 according to the World Health Organization [1]. The evidence suggests that children with higher socioeconomic status (SES) are more likely to be overweight (OW) or obese (OB) [2,3,4]. Ecuador is a country that has undergone rapid economic growth over the past decade, with two-thirds of the population moving from rural, agrarian settings to more urban, nonagricultural cities [5]. Urban cities provide such luxuries as convenience foods (i.e., fast food restaurants and refined bread and rice, etc.) and public transportation, which can lead to a less healthy environment, overweight status, and obesity. For example, Karkera et al. (2014) found that urban middle school youth (9–13 years) had significantly higher body mass index (BMI) and lower physical fitness and self-reported physical activity (PA) scores compared to their rural counterparts [6]. From this environmental shift and rising SES, excessive growth patterns have emerged in the Ecuadorean population [5]. The prevalence of overweight status/obesity in Ecuadorean adolescents rose from 18% in 2008–2009, to 23.1% and 29.5% in 2012 for Ecuadorian male and female adolescents, respectively [2,7]. Rates of OW/OB are higher in coastal (24.7%) compared to mountainous regions (17.7%) and in urban centers (Cuenca; 21.3%) compared to rural regions (Nabon; 14.7%) of the country [8]. While studies are revealing higher rates of obesity across the country of Ecuador, studies have not examined the factors that contribute to higher obesity rates.

Obesity is, in part, a consequence of chronic energy surplus associated with insufficient PA and/or excessive sedentary behavior (SB), a growing global issue. However, a recent international report from 36 countries found that 76% of Ecuadorean adolescents self-reported frequently participating in vigorous PA [9]. In contrast, Guthold et al. (2010) reported on adolescents across 34 countries, finding that 23.9% (boys) to 28.7% (girls) of the sample (*n* = 72,845) did not meet the PA recommendations. He further reported that >30% of adolescents in Ecuador spend at least three h/day in SB (i.e., watching TV and playing video games) in addition to sitting at school [10]. Like other developing countries, Ecuador is seeing a trend towards increased SB. Recent focus groups of Ecuadorean adolescents stated that they preferred SB, such as watching TV or playing video games, above PA pursuits [11]. These same Ecuadorean adolescents also stated that PA is secondary to schoolwork. Based on these statistics plus the success of well-controlled PA interventions [12,13], the Ecuador government stipulated an increase from two to five h/week of PA lessons in the national education system [14]. However, there have been no reports on the effectiveness of this initiative.

While measuring PA and SB in adolescents would help explain the relationship between lifestyle habits and weight status [9,15], methods of measuring these behaviors in previous research (i.e., self-report and diaries) are problematic in youth [16]. Objective methods of measuring PA are costly (activity monitors) or time-consuming (direct observation). However, fitness, a marker for cardiovascular health, is an indicator of previous PA habits, meaning that greater PA participation provokes positive changes in fitness levels [17]. In support of this relationship, the European Youth Heart Study revealed that adolescents (14–16 years) who perform at least 60 min of moderate-to-vigorous PA (MVPA) daily, the recommended dose for this age group, have three (females) to eight (males) times greater cardiovascular fitness compared to more sedentary adolescents [17]. With reports of Ecuadorean adolescents spending more time in SB and less time in PA, it is likely that poor physical fitness exists in this population [18].

As an increased proportion of the Ecuadorian population moves from rural, agricultural settings to urban cities, it is important to examine the factors that contribute to inactive lifestyles related to obesity. While the physical environment, such as access to recreational facilities and the walkability or safety of the neighborhood, is positively associated with youth PA participation levels [19], according to the social cognitive theory, the social environmental factors, such as parent or peer support, can also influence PA behavior [20]. Specifically, children with active parents are more likely to be physically active compared to children whose parents are not [21]. However, as previously stated, there is limited information about factors contributing to the increase in SB, reduction in PA, and the concomitant increase in obesity rates in Ecuadorean youth.

Therefore, the purpose of this study was to identify the physical (sex and weight status), environmental (location), and psychosocial (parental/peer support) factors that impact self-reported PA and measured physical fitness in Ecuadorean adolescents from the Pichincha and Loja provinces in Ecuador and determine the relationship among factors (PA and SB, fitness, weight status, and parental/peer support for PA) that may influence lifestyle behaviors.

## 2. Materials and Methods

### 2.1. Subjects

Between March and July 2015, a cross-sectional assessment of PA and SB participation, physical fitness, weight status, and perceived social (peer and parent) support for PA was conducted on adolescents from two locations within the Sierra region of Ecuador.

### 2.2. Sampling

Adolescents (13–18 years; *n* = 407) were recruited from four different schools: two schools from Pomasqui, a suburb of the Quito Metropolitan District (population: 28,910; elevation: 2600 m) within the Pichincha province, an urban, northern part of the Sierra region of Ecuador (*n* = 217); and two schools from the smaller town of Cariamanga (population: 13,311; elevation: 1950 m) within the Loja province, a rural, southern part of the Sierra region (*n* = 214). The local Ministry of Public Health in Ecuador approved the study to be conducted within the local schools. In accordance with the Declaration of Helsinki, and the institutional review boards of Ohio University and Pontificia Catholic University of Ecuador, a parent or legal guardian provided informed parental consent and each adolescent (<18 years) provided assent prior to participation. Those participants who were aged 18 years old provided their own informed consent to participate. Adolescents were excluded from the study if they had any physical, physiological, or other health issues (including pregnancy).

### 2.3. Anthropometrics

All measurements were performed by trained study personnel. For weight status classification, height to the nearest 0.1 cm and weight to the nearest 0.1 kg were used to calculate BMI (kg/m^2^), which was used to determine weight status according to the International Obesity Task Force (IOTF) standards. The IOTF uses growth curves that relate to the following adult cut-points: thinness 3 (<16 kg/m^2^); thinness 2 (<17 kg/m^2^); thinness 1 (<18.5 kg/m^2^); healthy weight (HW; <25 kg/m^2^); OW (<30 kg/m^2^); and OB (≥30 kg/m^2^). For this study, IOTF classifications of thinness 1–3 were collapsed as underweight (UW; <18.5 kg/m^2^), and OW and OB were collapsed as OW (≥25 kg/m^2^).

### 2.4. Resting and Fitness Measures

Prior to any assessments, each adolescent rested comfortably for ten minutes before trained study personnel manually measured resting heart rate (HR; beats/min). Adolescents then completed a 3-min step test to estimate physical fitness, expressed as immediate post-exercise HR (PostHR; beats/min) and estimated maximal oxygen consumption (estVO_2_max; mL/kg/min) [22]. To complete this test, adolescents were instructed to step at the rate of 24 steps/min on a stepper that was adjusted in height relative to individual stature; step height ranged from 10 to 16 inches in 2-inch increments. A metronome was set at a rate of 96 beats/min to count the four portions of a complete step: up, up, down, down. Verbal encouragement throughout the test helped each adolescent maintain the proper cadence for the entire three minutes. Immediately post-test (<5 s), the adolescent was instructed to sit down on the step, and a trained member of study personnel manually measured PostHR for 15 s. Previous research has revealed a strong relationship (*r* = 0.81 to 0.96) between measured VO_2_max and estVO_2_max from a 3-min step test in youth [22]. Submaximal oxygen consumption (VO_2_) was calculated from the PostHR, step height, and cadence using a child-specific metabolic equation [23]:

[VO_2_ (mL/kg/min) = (0.2 × step rate) + (2.4 × step height (cm) × step rate) + 3.5] (1)

EstVO_2_max was calculated based on the linear relationship between HR and VO_2_, calculating the PostHR as a percentage of the adolescents predicted maximal HR (estimated HRmax = 208 − (0.7 × (age)) [24]. EstVO_2_max (Table 1 and Table 2) was used to classify physical fitness levels according to age- and sex-specific classifications (very poor, poor, fair, good, excellent, and superior) [25].

### 2.5. Questionnaire

Following the step test, adolescents completed a modified version of the School Physical Activity and Nutrition (SPAN) questionnaire on an iPad mini. The questionnaire, validated for monitoring students’ PA and SB habits, was adapted to be more culturally relevant for Ecuadorean adolescents and then translated into Spanish [26]. Trained Spanish-speaking study personnel helped each adolescent as they completed the questionnaire, which asked them to self-report their weekly PA and SB habits and perceived social support for PA. Specifically, they were asked to report how many days per week they typically participated in MVPA for at least 60 min (0–7 days/week) and in strength training (0–7 days/week), as well as how many school and non-school sports they participated in during the past 12 months (0–3+ teams/year) and whether they participated in any organized PA lessons, such as karate or dance (0 = no; 1 = yes). As an indicator of the amount of time spent in SB, adolescents were asked to report separately how much time (0–6+ h/day) they typically spent watching TV, working or playing on the computer, and playing video games. To assess the level of social support for PA participation or fitness, adolescents rated their perceived parents’ value of and participation in PA. Similar questions were asked about the social support for PA from their peers. For example, adolescents reported how they perceived their parents would respond if they stopped exercising by using a Likert scale ranging from “*not at all (upset)*” (0) to “*very upset*” (3), while perceived parent and peer support questions (i.e., encouraged participation or participated with them) were answered using a Likert scale ranging from “*never*” (0) to “*always*” (4).

### 2.6. Data Analysis

Descriptive statistics are reported for the overall sample and stratified by main effects: demographics (sex, location, and school) and physical condition (weight status and fitness classification). Means (±SD) are reported for continuous variables and frequencies (%) are reported for categorical variables. Composite scores were calculated for PA participation (PA Comp = number of days/week they engaged in 60 min of MVPA + number of sessions/week of strength training; range = 0–14 sessions/week), sport participation (Sport Comp = number of school sports per year + number of non-school sports per year + organized PA lesson participation; range = 0–7 teams/year), SB (SB Comp = TV viewing + Computer use + Video Game play; range = 0–18 h/day), Parent Support for PA (Parent Comp = Parent’s PA value + Parent’s PA Influence + Parent’s PA participation and practice; score range: 0–15), and Peer Support for PA (Peer Comp = Friends’ PA value + Friends’ PA participation and practice: score range: 0–12). *T*-tests were used to assess differences across sex (male vs. female) and location (rural vs. urban regions of Ecuador) and one-way analysis of variance (ANOVA) was used to assess differences across schools (two schools in the urban region + two schools in the rural region), weight status (UW, HW, and OW), and fitness level (very poor, poor, fair, good, excellent, and superior). Relevant interactions of the main effects were assessed using a Tukey–Kramer post hoc adjustment to prove the locus of significant interactions. Logistic regression analyses were conducted with physical fitness and PA and SB participation as dependent variables and the composite scores as the predictor alone and by sex, location, and weight status. Pearson r correlations were used to assess the relationships among all predictor and outcome variables. All statistics were performed using SAS 9.4 and significance was set at *p* < 0.05.

## 3. Results

### 3.1. Demographics

General characteristics are presented overall and by demographics (sex, location and school) in Table 1 and by status (weight status and physical fitness) in Table 2. As expected, males were taller and heavier than females. Unexpectedly, adolescents from urban Sierra schools (Quito) were older, taller, and heavier than adolescents from rural Sierra schools (Cariamanga). To illustrate the range in demographics across schools, the youngest sample from Cariamanga was on average 1.4 years younger (adjusted *p* < 0.0001), 5.2 cm shorter (adjusted *p* = 0.03), and 10.4 kg lighter (adjusted *p* < 0.0001) than the oldest sample from Quito. It is important to note that UW adolescents were significantly younger (14.4 ± 1.5 years) than HW or OW adolescents (15.3 ± 1.6 years and 15.6 ± 1.4 years, respectively).

### 3.2. Physical Fitness

**Figure 1:**
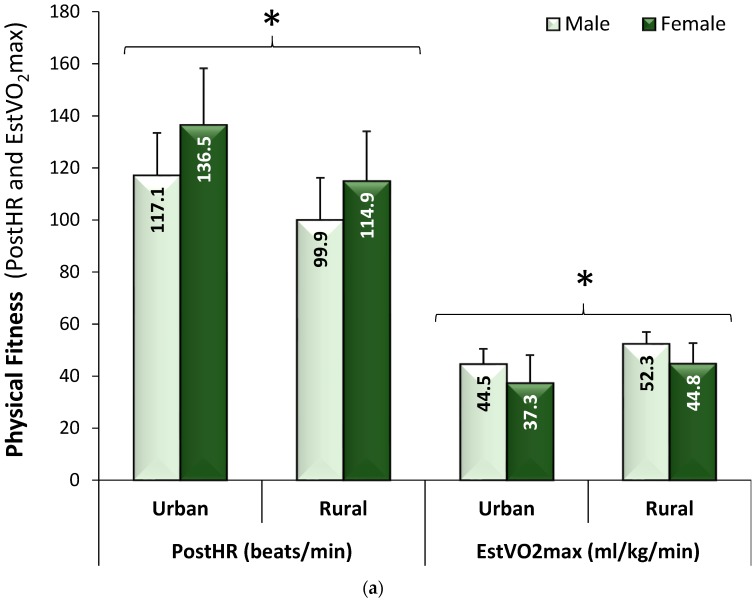

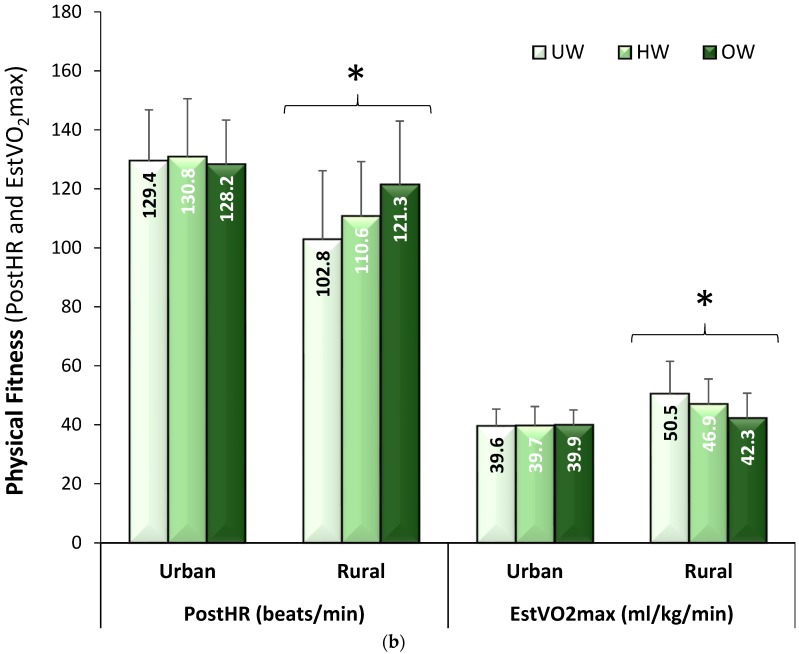
(**a**) Physical fitness levels for male and female adolescents by location, which illustrates that females had consistently lower fitness levels than males, regardless of location; (**b**) Physical fitness levels for underweight (UW), healthy weight (HW), and overweight/obese (OW) adolescents by location, which illustrates that physical fitness levels were significantly higher in UW and HW adolescents compared to OW adolescents only in the rural (southern) Sierra region with no difference in physical fitness across weight status in the urban (northern) Sierra region (Means ± SD). * adjusted *p* < 0.0001. PostHR: immediate post-exercise heart rate; EstVO_2_max: estimated maximal oxygen consumption.

A lower resting HR and Post HR and a higher estVO_2_max were considered as evidence of greater physical fitness. Those who were unable to complete the step test, due to injury, were removed from all analyses (*n* = 2). Overall, males and rural adolescents were more physically fit than females and urban adolescents (Table 1 and Table 2), with significant sex by location interactions (Figure 1a). Further, UW and HW adolescents from the rural Sierra schools were more physically fit than UW and HW adolescents from the urban Sierra schools, while there was no effect of location for OW adolescents (Figure 1b). Closer investigation found estVO_2_max and PostHR differed significantly across all the schools and that PA (β = 0.45, *t* = 3.73, *p* = 0.0002), sport (β = 0.66, *t* = 2.32, *p* = 0.02), and SB (β = −0.57, *t* = −3.47, *p* = 0.0006) composite scores significantly predicted the adolescents’ physical fitness levels.

### 3.3. Physical Activity Habits

PA, sport, and SB data are also reported in Table 1 and Table 2. Using the current PA guidelines from the World Health Organization [27], only 8.4% of the adolescents, more so in the rural region (12.4%) than the urban region (4.4%), reported participating in 60 min of daily MVPA; 29.0% reported at least three days/week of strength training similarly across regions, and 30.2% reported spending ≤2 h/day in SB (Table 3). Males reported participating in 0.76 more days/week of MVPA for at least 60 min, 1.2 more days/week of strength training, and more organized sports or PA lessons (i.e., gymnastics, karate) each year compared to females, but males also reported spending over 0.5 h/day more time playing video games (adjusted *p* < 0.0001) than females. More females met the SB recommendations (≤2 h/day) than males. HW adolescents were more likely to meet the PA and SB recommendations for their age compared to OW adolescents, while UW adolescents reported spending significantly more time playing video games compared to HW or OW adolescents. There was no relationship found between PA participation and obesity, whereas only video game use was weakly and negatively associated with obesity status.

Location had no effect on self-reported PA or sport participation, but did have a significant effect on SB, such that urban adolescents spent more time watching TV or using computers compared to rural adolescents. Differences in PA and SB participation persisted between schools, especially in the rural schools. Greater percentage of the adolescents from the rural schools met both PA and SB recommendations, with the greatest percentage of adolescents attending Rural1 school being sufficiently active. It is important to note that individual components of self-reported PA and SB participation (except for video game use) were weakly correlated with physical fitness with correlation coefficients ranging from *r* = −0.18 to 0.20.

### 3.4. Parent and Peer Support

Composite scores were used to compare adolescents’ perceptions of peer and parent support for PA participation overall and by sex, fitness status, weight status, and location. Males (8.7 ± 2.7) reported greater peer influence on PA than females (7.5 ± 3.1) on a scale of 0–15 (*p* = 0.0004), while there were no differences in peer or parent influence on PA reported by sex, fitness, weight or location. Parent and peer influence on PA were positively correlated with self-reported time spent in MVPA, while only peer influence positively correlated with physical fitness and parent influence was negatively correlated with self-reported TV viewing (Table 4).

## 4. Discussion

This study assessed the self-reported PA and SB participation, peer and parent support for PA, and measured physical fitness of Ecuadorian adolescents from two different regions of Ecuador: the urban Pichincha (Quito) and rural Loja (Cariamanga) provinces. We examined the impact of physical, environmental, and psychosocial factors on PA and SB participation and the relationship among these same variables.

According to IOTF standards, 12.3% (boys: 6.9%; girls: 14.8%) were classified as OW, which did not differ by region but was significantly different across individual schools. Ochoa-Aviles et al. (2012) reported higher rates of obesity, with 18% of Ecuadorian adolescents being classified as OW, while Freire et al. (2014) reported ever higher OW rates in 12–19 year old adolescents as part of the Ecuadorian National Health and Nutrition Survey (boys: 23.1%; girls: 29.5%) [2,7]. Previous research also noted that OW rates were inconsistent across regions of Ecuador, which was not observed in the current study. Lower OW rates in the current study may be explained, in part, by recruitment bias. Specifically, inclusion of a fitness assessment component (e.g., step test) in the testing protocol may have dissuaded some less fit and/or more OW adolescents from participating in the study. Neither of the two studies cited above incorporated such a fitness assessment component, but this does not explain the lack of regional variations in weight status in this study.

While the OW rates were low, self-reported SB participation, a major contributor to obesity [28], was high, with 70% of the adolescents considered sedentary by reporting >2 h/day of SB, 12.9% more so in the urban than the rural Sierra region. This predicts that rates of OW will increase over time, forecasted by the increased obesity and SB in the urban areas. In previous research, Guthold et al. (2010) classified only 30% of Ecuadorian adolescents (*n* = 1267; 13–15 years) as sedentary; however, they used ≥3 h/day of screen-time as a cut-point for sedentary, which may explain the incongruences between these Ecuador-based studies [10]. Andrade et al. (2015) also used the same screen-time cut-point as a surrogate for SB and reported 69% of adolescents (*n* = 1440; 12.8 ± 0.8 years) spending ≥3 h/day in SB [12]. Using this same cut-off point in the current study reduces the percentage of adolescents classified as sedentary from 70% (*n* = 284) to 65.6% (*n* = 267) of the sample. It is well documented that SB is associated with greater risk of obesity-related diseases, such as type 2 diabetes, cancer, and cardiovascular disease, even in youth [29]. Reducing SB is one of the solutions to combating obesity and these obesity-related diseases [12,30]. However, we found no associations between OW rates and overall SB participation (composite score), emphasized by the fact that greater levels of SB reported in the urban Sierra region were not related to higher obesity levels.

While reducing SB is important for healthy growth trajectories, insufficient PA participation is independently association with obesity in youth. In support, Andersen et al. (1998) found youth who participate in the most PA are least likely to be OW [31]. This trend holds true today in the United States and other countries, including Ecuador. For example, Abril et al. (2013) found that low PA participation was a significant contributor to childhood obesity (6–9 years) in Cuenca, Ecuador [4]. In the current study, only 8% (*n* = 32) of the 407 adolescents reported at least 60 min of daily MVPA, which was similar between regions and across individual schools, and there was no association between PA participation and weight status. Using the composite score, combining number of days adolescents engaged in 60 min of MVPA with the number of strength training sessions/week, the percentage engaged in ≥7 sessions/week rose to 50.4% (*n* = 205), but this score does not specify if these sessions were on different days of the week. Regardless of how the data is combined, the percentage of those meeting the PA guidelines is lower compared to other Ecuadorian research. For example, Ortega et al. (2008) reported that 61 to 70% of 14–16 year old male and female Ecuadorean adolescents, respectively (*n* = 472), met the PA guidelines for adolescents [17]. The differences between this and the current study may be related to the method of measuring PA (self-report vs. objective monitoring). While self-reported PA measures are subject to over-estimations due to social desirability bias, large epidemiologic studies still rely on this method. For example, a collection of national Active Health Kids Report Cards used self-reported data to determine the percentage of youths meeting the PA guidelines, with grades ranging from B (60–79%) to F (<20%) across 15 different countries; unfortunately, Ecuador was not included [27]. However, using this grading system, adolescents from the current study would earn an “F” (<20%) for overall PA participation. Although it is important to note, our findings did agree with previous research in that boys were more physically active than girls [10,17].

Relationships among PA and SB participation and physical fitness have been reported in the literature. Analysis of the 2012 National Health and Nutrition Examination Study (NHANES) data (*n* = 1576), youth with low PA participation and high SB had greater odds of having lower physical fitness levels. Further, Moore et al. (2013) found that SB and PA participation partially but significantly explain variances in physical fitness in middle school youth (*n* = 285), such that adolescents with higher SB and lower PA participation had significantly lower fitness [32]. In contrast, in the current study, there were weak relationships between PA and SB individual and composite scores and physical fitness. Specifically, we found a weak but positive relationship between the number of MVPA sessions/week and physical fitness (*p* = 0.0015) and a weak but negative relationship between TV use (*p* = 0.0003) and computer use (*p* = 0.0007) and physical activity. While 72% of the adolescents had a physical fitness level classified as “good” to “excellent,” with no difference by location, they did not report the expected high levels of PA and low levels of SB participation as seen in previous research, plus there were no significant differences in self-reported MVPA participation across physical fitness classifications (Table 2). This level of physical fitness was also higher than that reported previously by Andrade et al. (2014), who reported 59% of Ecuadorean adolescents (*n* = 648; 11–15 years) had “poor” fitness levels [18]. The inconsistencies between the current and previous research may be due to the aforementioned recruitment bias, while inconsistencies within the current analyses could be related to small samples sizes in some of the sample subgroups (e.g., poor physical fitness, *n* = 8).

The final aim of this study was to assess the psychosocial factors, namely the perceived parent and peer support for PA, in Ecuadorean adolescents from different regions of the country. We found inconsistent evidence that perceived social support for PA positively impacted PA and SB participation or physical fitness, with no difference across regions. There was no association between social support for PA and weight status, and peer support was more important than parent support for positively impacting PA participation and physical fitness. Social support for PA can arise from modeling a behavior, encouraging participation, and attending and/or providing transportation to and from sporting or activity events. High levels of peer support for PA have been related to both initiating and maintaining PA [33,34]. In a review of the literature on this topic, Beets et al. (2010) found consistent reports that both peer and parent support for PA had mainly positive effects on children’s PA levels [34]. This support for PA ranged from “encouraging their child to go outside” (intangible support) to “buy(ing) sports clothing/equipment” (tangible support). The questions used in the current study to determine social support included both tangible (i.e., participates in PA with the adolescent) and intangible (i.e., watches the adolescent’s activity or sporting event) support questions for both parent and peer. However, our findings only support the literature that peer, not parent, support for PA is important for promoting PA participation and physical fitness. Neither parent or peer support for PA were important for reducing SB participation, except for TV use. Further research is needed to determine if other psychosocial factors may be contributing to the PA and SB participation and physical fitness in this population.

While this study sample was large, included empirically measured physical fitness, and used two different regions of Ecuador, the study does have limitations. For example, expanding to include more sites throughout the country to better represent the whole, diverse population of Ecuador may increase the understanding of the relationships between PA habits and obesity. Also, adolescents were asked to self-report how many days per week they typically engaged in 60 min of MVPA and to report their typical daily screen time use, rather than objectively monitoring these behaviors. The questionnaire did not provide actual amounts of time spent in PA or SB, which may have produced stronger associations among PA and SB participation, physical fitness, and weight status. While using objective monitors to detect and record time spent in all PA intensities, including SB, is planned for future studies, there is ample evidence that objective monitors tend to misclassify PA intensity, which can result in significant over- or under-estimation of the amount of time spent in MVPA and SB [35]. The weak associations between self-reported PA participation and measured physical fitness, used in this study as a surrogate of PA participation, further accents the need for better measurement of PA participation. While previous research reports strong relationships between PA participation and physical fitness, others have found no relationship. For example, Karkera et al. (2014) found no relationship between self-reported PA participation and estimated physical fitness from a 20-m shuttle run in a cross-sectional study of both rural (*r* = −0.075) and urban (*r* = 0.056) Indian youth [6]. While the 20-m shuttle run and the 3-min step test are validated, age-appropriate methods for estimating maximal aerobic fitness (VO_2_max), they can be another source of error. For examples, a study comparing the 3-min step test to measured VO_2_max with a traditional treadmill test reported an standard error of estimate of ±5.7 mL/kg/min (R^2^ = 0.64), which could result in misclassification of physical fitness status [22]. Another confounding factor that may have influenced the results which was not measured in this study was the socioeconomic status of the two regions of Ecuador. With Cariamanga being more rural as compared to a suburb of Quito, differences in affluence could affect the availability of TV and video games, which undoubtedly could have reduced this impact on SB participation.

## 5. Conclusions

In conclusion, this study demonstrated the need to promote increased PA and decreased SB participation in both regions of Ecuador in order to help combat the growing obesity problem. However, more research is needed to understand the factors contributing to obesity, as other factors, such as dietary intake, may be contributing to this problem other than activity or inactivity levels in this sample. Plans to investigate the impact of self-reported dietary intake on obesity are intended in future analyses in an attempt to more fully understand the driving forces behind Ecuador’s obesity problem.

## Figures and Tables

**Table 1:** Participant characteristics overall and by sex, location, and school. Means ± SD.

Variables	Total Sample	Sex		Location		School			
		Male	Female	Urban	Rural	Urban 1	Urban 2	Rural 1	Rural 2
Sample Size	407	130	277	205	202	86	119	106	96
**Demographics**									
Age (years)	15.1 ± 1.6	15.1 ± 1.6	15.1 ± 1.6	15.4 ± 1.6 †	14.8 ± 1.5 †	16.0 ± 1.7 ‡	14.9 ± 1.4 ‡	15.1 ± 1.5 ‡	14.6 ± 1.4 ‡
Height (cm)	155.8 ± 7.9	160.7 ± 9.7 ‡	153.5 ± 5.6 ‡	157.3 ± 8.0 †	154.3 ± 7.5 †	158.8 ± 7.3 *	156.2 ± 8.4 *	154.9 ± 8.4 *	153.6 ± 6.2 *
Weight (kg)	52.0 ± 13.4	53.9 ± 16.3	51.2 ± 11.8	54.4 ± 16.1 †	49.6 ± 9.5 †	60.2 ± 20.4 ‡	50.2 ± 10.2 ‡	49.4 ± 10.0 ‡	49.9 ± 8.9 ‡
BMI (kg/m^2^)	21.3 ± 4.9	20.6 ± 5.4 *	21.7 ± 4.6 *	21.9 ± 6.1 +	20.8 ± 3.3 +	23.9 ± 8.1 ‡	20.5 ± 3.5 ‡	20.5 ± 3.3 ‡	21.1 ± 3.2 ‡
IOTF BMI (%ile)	49.0 ± 29.5	39.9 ± 28.9 ‡	53.3 ± 28.9 ‡	47.3 ± 29.6	50.7 ± 29.4	46.3 ± 27.8 *	48.1 ± 31.0 *	45.7 ± 29.4 *	56.3 ± 28.5 *
**Physical Fitness**									
PostHR (beats/15 s)	30.1 ± 5.6	27.2 ± 5.2 ‡	31.4 ± 5.2 ‡	32.5 ± 4.7 ‡	27.6 ± 5.3 ‡	31.0 ± 4.5 ‡	33.7 ± 4.4 ‡	26.4 ± 4.9 ‡	28.8 ± 5.4 ‡
EstVO_2_max (mL/kg/min)	43.4 ± 8.8	48.3 ± 9.4 ‡	41.1 ± 7.5 ‡	39.7 ± 6.1 ‡	47.1 ± 9.6 ‡	41.7 ± 6.3 ‡	38.2 ± 5.6 ‡	48.9 ± 9.3 ‡	45.2 ± 9.6 ‡
**Physical Activity Questions**									
PA Comp (0–14)	6.7 ± 3.6	8.1 ± 3.8 ‡	6.0 ± 3.2 ‡	6.7 ± 3.4	6.6 ± 3.7	6.5 ± 3.7	6.8 ± 3.2	6.5 ± 3.8	6.8 ± 3.7
MVPA (days/week)	2.9 ± 2.0	3.4 ± 2.2 †	2.6 ± 1.9 †	2.7 ± 1.8	3.0 ± 2.2	2.7 ± 2.1	2.7 ± 1.6	2.9 ± 2.2	3.2 ± 2.1
Strength (sessions/week)	1.9 ± 2.0	2.7 ± 2.4 ‡	1.5 ± 1.7 ‡	1.9 ± 1.9	1.9 ± 2.2	1.8 ± 2.1	2.0 ± 1.7	1.9 ± 2.3	1.8 ± 2.0
Sport Comp (0–7)	1.6 ± 1.5	1.8 ± 1.7	1.5 ± 1.4	1.7 ± 1.6	1.7 ± 1.5	1.7 ± 1.6	1.6 ± 1.5	1.5 ± 1.5	1.7 ± 1.5
School Teams (#/year)	0.6 ± 0.8	0.8 ± 0.9 *	0.6 ± 0.8 *	0.6 ± 0.8	0.6 ± 0.8	0.8 ± 0.8	0.6 ± 0.8	0.7 ± 0.8	0.6 ± 0.8
Non-School Teams (#/year)	0.6 ± 0.8	0.8 ± 0.9 *	0.6 ± 0.8 *	0.7 ± 0.9	0.6 ± 0.8	0.6 ± 0.9	0.7 ± 0.8	0.6 ± 0.8	0.6 ± 0.8
Organized Lessons (#/year)	0.4 ± 0.5	0.3 ± 0.5 *	0.4 ± 0.5 *	0.4 ± 0.5	0.4 ± 0.5	0.3 ± 0.5 *	0.4 ± 0.5 *	0.3 ± 0.5 *	0.5 ± 0.5 *
SB Comp (0–18)	4.0 ± 2.7	4.5 ± 2.9 +	3.8 ± 2.5 +	4.7 ± 2.8 ‡	3.3 ± 2.3 ‡	4.8 ± 2.7 ‡	4.7 ± 2.8 ‡	3.3 ± 2.2 ‡	3.4 ± 2.4 ‡
TV viewing (h/day)	2.0 ± 1.4	1.9 ± 1.4	2.1 ± 1.4	2.3 ± 1.5 ‡	1.7 ± 1.3 ‡	2.5 ± 1.6 †	2.2 ± 1.4 †	1.8 ± 1.1 †	1.7 ± 1.4 †
Computer Use (h/day)	1.8 ± 1.6	1.9 ± 1.7	1.7 ± 1.6	2.2 ± 1.8 ‡	1.4 ± 1.3 ‡	2.2 ± 1.7 ‡	2.3 ± 1.8 ‡	1.2 ± 1.3 ‡	1.6 ± 1.4 ‡
Video Game (h/day)	0.4 ± 0.9	0.8 ± 1.2 ‡	0.2 ± 0.7 ‡	0.5 ± 1.0	0.4 ± 0.8	0.4 ± 0.9	0.5 ± 1.1	0.4 ± 0.8	0.3 ± 0.6

BMI: body mass index; IOTF: International Obesity Task Force weight classifications; HR: heart rate; PostHR: immediate post step test HR; estVO_2_max: estimated maximal oxygen consumption; PA: physical activity; SB: sedentary behavior; MVPA: moderate-to-vigorous PA; PA Comp: composite score combining self-reported number of sessions of MVPA and strength training per week; Sport Comp: composite score of self-reported number of school and non-school sports teams and PA lessons per year; SB Comp: composite score of self-reported weekly TV, computer and video game use. Significance set at adjusted *p* < 0.05. * *p* < 0.05; + *p* < 0.01; † *p* < 0.001; ‡ *p* < 0.0001.

**Table 2:** Participant characteristics by weight status and physical fitness classification. Means ± SD.

Variables	IOTF Standards			Physical Fitness					
	UW	HW	OW	Very Poor	Poor	Fair	Good	Excellent	Superior
Sample Size	112	245	50	8	17	76	116	62	128
**Demographics**									
Age (years)	14.4 ± 1.5 ‡	15.3 ± 1.6 ‡	15.6 ± 1.4 ‡	14.6 ± 1.7	15.3 ± 1.8	15.2 ± 1.6	15.5 ± 1.5	14.9 ± 1.6	14.9 ± 1.6
Height (cm)	154.3 ± 8.4 *	156.7 ± 7.5 *	155.2 ± 8.1 *	157.3 ± 12.4 *	156.0 ± 8.5 *	155.7 ± 7.8 *	157.5 ± 8.0 *	156.4 ± 8.5 *	154.0 ± 6.9 *
Weight (kg)	43.8 ± 13.3 ‡	52.9 ± 11.3 ‡	66.2 ± 9.5 ‡	50.1 ± 14.9	52.1 ± 11.6	52.0 ± 14.3	53.9 ± 11.9	52.4 ± 13.3	50.3 ± 14.3
BMI (kg/m^2^)	18.3 ± 5.2 ‡	21.5 ± 3.9 ‡	27.4 ± 2.6 ‡	20.0 ± 4.3	21.2 ± 3.2	21.4 ± 5.7	21.7 ± 4.4	21.3 ± 5.0	21.1 ± 5.1
IOTF BMI (%ile)	14.7 ± 10.7 ‡	55.4 ± 19.8 ‡	94.5 ± 4.0 ‡	47.4 ± 41.2	56.7 ± 30.0	43.5 ± 30.3	52.0 ± 31.5	47.7 ± 28.3	49.2 ± 26.9
**Physical Fitness**									
PostHR (beats/15 s)	29.3 ± 6.1	30.2 ± 5.4	31.0 ± 4.9	37.6 ± 2.8 ‡	36.7 ± 4.1 ‡	34.6 ± 4.2 ‡	32.0 ± 3.7 ‡	29.1 ± 3.4 ‡	24.8 ± 3.8 ‡
EstVO_2_max (mL/kg/min)	44.6 ± 10.2	43.2 ± 8.4	41.4 ± 7.4	33.0 ± 1.5 ‡	34.2 ± 3.9 ‡	36.9 ± 4.6 ‡	40.2 ± 5.2 ‡	44.0 ± 5.9 ‡	51.6 ± 8.5 ‡
**Physical Activity Questions**									
PA Comp (0–14)	6.4 ± 3.3	6.9 ± 3.7	6.3 ± 3.3	5.0 ± 4.8	6.5 ± 4.0	7.0 ± 3.0	6.8 ± 3.6	6.5 ± 3.6	6.6 ± 3.7
MVPA (days/week)	2.8 ± 2.0	2.9 ± 2.0	2.6 ± 2.0	2.0 ± 2.3	2.8 ± 1.8	3.0 ± 1.8	2.8 ± 2.0	2.8 ± 2.0	3.0 ± 2.1
Strength (sessions/week)	1.5 ± 1.6	2.1 ± 2.2	1.7 ± 1.8	1.6 ± 2.3	1.8 ± 2.4	1.8 ± 1.7	2.0 ± 2.1	1.8 ± 2.0	1.8 ± 2.2
Sport Comp (0–7)	1.7 ± 1.7	1.6 ± 1.5	1.4 ± 1.3	1.3 ± 1.6	1.2 ± 1.1	1.6 ± 1.6	1.6 ± 1.5	1.8 ± 1.6	1.6 ± 1.6
School Teams (#/year)	0.8 ± 1.0	0.6 ± 0.8	0.5 ± 0.8	0.5 ± 1.1	0.5 ± 0.6	0.6 ± 0.8	0.6 ± 0.8	0.7 ± 1.0	0.7 ± 0.8
Non-School Teams (#/year)	0.6 ± 0.9	0.7 ± 0.8	0.5 ± 0.7	0.5 ± 0.8	0.5 ± 0.7	0.6 ± 0.8	0.7 ± 0.9	0.7 ± 0.9	0.6 ± 0.8
Organized Lessons (#/year)	0.3 ± 0.5	0.4 ± 0.5	0.4 ± 0.5	0.3 ± 0.5	0.2 ± 0.4	0.3 ± 0.5	0.4 ± 0.5	0.4 ± 0.5	0.4 ± 0.5
SB Comp (0–18)	4.1 ± 2.7	4.1 ± 2.6	3.8 ± 2.6	6.4 ± 5.0 ‡	5.6 ± 3.5 ‡	4.6 ± 2.7 ‡	4.1 ± 2.4 ‡	4.5 ± 2.6 ‡	3.1 ± 2.3 ‡
TV viewing (h/day)	2.0 ± 1.4	2.0 ± 1.4	2.0 ± 1.6	2.4 ± 2.1 *	2.4 ± 1.4 *	2.2 ± 1.5 *	1.9 ± 1.3 *	2.3 ± 1.5 *	1.7 ± 1.3 *
Computer Use (h/day)	1.7 ± 1.6	1.9 ± 1.7	1.8 ± 1.5	2.8 ± 2.8 †	2.8 ± 2.1 †	2.0 ± 1.8 †	2.0 ± 1.6 †	2.0 ± 1.4 †	1.3 ± 1.3 †
Video Game (h/day)	0.6 ± 1.1 *	0.4 ± 0.8 *	0.2 ± 0.5 *	1.1 ± 1.5	0.6 ± 1.5	0.6 ± 0.9	0.3 ± 0.8	0.5 ± 1.0	0.3 ± 0.8

BMI: body mass index; IOTF: International Obesity Task Force weight classifications; HR: heart rate; PostHR: immediate post step test HR; estVO_2_max: estimated maximal oxygen consumption; PA: physical activity; SB: sedentary behavior; MVPA: moderate-to-vigorous PA; PA Comp: composite score combining self-reported number of sessions of MVPA and strength training per week; Sport Comp: composite score of self-reported number of school and non-school sports teams and PA lessons per year; SB Comp: composite score of self-reported weekly TV, computer and video game use; UW: underweight; HW, healthy weight; OW: overweight or obese; Significance set at adjusted *p* < 0.05 (Tukey–Kramer). * *p* < 0.05; † *p* < 0.001; ‡ *p* < 0.0001.

**Table 3:** Frequency of adolescents meeting the physical activity and sedentary guidelines. Number (%).

Variables	Total	Sex		IOTF Standards			Location		School			
		Male	Female	UW	HW	OW	Urban	Rural	Urban 1	Urban 2	Rural 1	Rural 2
Sample Size (N)	407	130	277	112	245	50	205	202	86	119	106	96
PA Comp Score	205	82	123	57	125	23	102	103	37	65	49	54
	(50.4)	(63.1)	(44.4)	(53.9)	(51.0)	(46.0)	(49.8)	(51.0)	(43.0)	(54.6)	(46.2)	(56.3)
MVPA (≥7 days/week)	34	20	14	8	23	3	9	25	6	3	17	8
	(8.4)	(15.4)	(5.1)	(7.1)	(9.4)	(6.0)	(4.4)	(12.4)	(7.0)	(2.5)	(16.0)	(8.3)
Strength (≥3 days/week)	118	57	61	26	79	13	59	59	25	34	30	29
	(29.0)	(43.9)	(22.0)	(23.2)	(32.2)	(26.0)	(28.8)	(29.2)	(29.1)	(28.6)	(28.3)	(30.2)
SB Comp Score	123	33	90	33	73	17	44	79	16	28	42	37
	(30.2)	(25.4)	(32.5)	(29.5)	(29.8)	(34.0)	(21.5)	(39.1)	(18.6)	(23.5)	(39.6)	(38.5)
≤2 h TV viewing	268	93	175	75	162	31	121	147	49	72	78	69
	(67.3)	(72.7)	(64.8)	(68.8)	(66.9)	(66.0)	(60.8)	(73.9)	(57.0)	(63.7)	(75.0)	(72.6)
≤2 h Computers	278	85	193	80	163	35	123	155	51	72	85	70
	(73.5)	(66.9)	(76.9)	(76.9)	(71.2)	(77.8)	(65.1)	(82.0)	(64.6)	(65.5)	(84.2)	(79.6)
≤2 h Video Games	389	116	273	104	235	50	192	197	80	112	102	95
	(96.1)	(90.6)	(98.6)	(92.9)	(96.7)	(100.0)	(94.1)	(98.0)	(93.0)	(94.9)	(97.1)	(99.0)

PA: physical activity; SB: sedentary behavior; MVPA: moderate-to-vigorous PA; IOTF: International Obesity Task Force weight classifications; UW: underweight; HW: healthy weight; OW: overweight or obese; Urban: Northern Sierra (Quito) region; Rural: Southern Sierra (Cariamanga) region; PA Comp Score: combination of those meeting ≥60 min/week of MVPA and strength training guidelines; SB Comp Score: combination of those meeting the sedentary guidelines of ≤2 h/week of screen time.

**Table 4:** Correlation matrix for physical fitness, physical activity participation and sedentary behaviors.

Variables	Weight Status	Physical Fitness		PA Participation			SB Participation			
	BMI %ile	1a	1b	PA Comp	2a	2b	SB Comp	3a	3b	3c
1. Physical Fitness										
a. PostHR (b/min)	0.10 *									
b. EstVO_2_max (mL/kg/min)	−0.11 *	−0.94 **								
2. PA Composite Score	0.01	−0.17 *	0.18 *							
a. MVPA 60-min	−0.01	−0.15 *	0.16 *	0.78 **						
b. Strength Training	0.05	−0.16 *	0.19 *	0.85 **	0.48 **					
3. SB Composite Score	−0.03	0.19 *	−0.17 *	0.10 *	0.04	0.05				
a. TV Use	−0.01	0.18 *	−0.18 *	−0.16 *	−0.14 *	−0.20 *	0.68 **			
b. Computer Use	0.04	0.20 *	−0.17 *	0.19 *	0.13 *	0.17 *	0.80 **	0.23		
c. Video Game Use	−0.13 *	−0.05	0.07	0.17 *	0.06	0.14 *	0.47 **	0.03	0.21	
4. Parent Support Comp Score	−0.00	−0.07	0.06	0.12 *	0.15 *	0.02	−0.06	−0.16 *	0.00	0.00
5. Peer Support Comp Score	−0.04	−0.12 *	0.14 *	0.15 *	0.19 *	0.09	−0.03	−0.01	−0.03	−0.05

BMI %ile: body mass index percentiles; PA: physical activity; MVPA: moderate-to-vigorous PA; SB: sedentary behavior; PA Comp: composite score combining self-reported number of sessions of MVPA and strength training per week; SB Comp: composite score of self-reported weekly TV, computer and video game use; PostHR: immediate post exercise heart rate in beats/min; EstVO_2_max: estimated maximal aerobic capacity; * *p* < 0.05; ** *p* < 0.001.
